# Letter to the readers: proposal for a new year's resolution

**DOI:** 10.3205/zma001305

**Published:** 2020-02-17

**Authors:** Wolf Hautz

**Affiliations:** 1 Inselspital Bern, Universitäres Notfallzentrum, Bern, Switzerland

## Editorial

Dear reader, 

Many colleagues have contributed for 2020 to start with this new edition of the Journal of Medical Education (JME). Many authors have written and revised manuscripts, designed and carried out the projects or studies they report on, and collected and analysed the data. I believe that this effort has been worthwhile, and we owe the authors a debt of gratitude for sharing their findings with us. 

In addition to the authors' obvious contribution, 2 editors in chief, 33 editors and a lot of reviewers have made this edition of JME possible with great (and free) commitment. In the editorial office, Mrs. Hespelein has coordinated the work of all these colleagues with infinite patience, reminding them and, as a result, completing a successful new edition of JME. 

But this work was only the tip of the iceberg. The authors, reviewers and editors of the rejected articles have also invested effort and expense, which, arguably, is much more difficult to quantify because only accepted articles are assigned to a specific issue. A simple estimate of this effort can be made by looking at the rejection rate, which is currently just over 50% and rising at the JME. The actual expenditure for an issue of the JME is therefore at least twice as high as calculated based on the published articles. 

Other journals in our field have similar, and in some cases significantly higher rejection rates (currently 87% for Advances in Health Science Education). Assuming that on average about every third article is accepted and every manuscript is reviewed by at least two reviewers (and one editor), every published article requires the free (and mostly invisible) commitment of at least 9 colleagues. With an average of less than 4 authors per article [1], it follows that for each article I publish, I need to review at least two articles by colleagues in order to keep the system working in the medium term. 

From my work as co-editor of the JME, as associate editor at BMC Medical Education, from an editorial internship at Medical Education and from many conversations with colleagues, I have noticed that it is becoming increasingly difficult to find reviewers for submitted manuscripts and that the work is distributed on fewer and fewer shoulders. Sometimes we ask 10 or 15 different colleagues (my personal record is 26) for an expert opinion before two commit.

This has various consequences: As editors, we increasingly rely on the opinion of a few reviewers. Their actual expertise becomes increasingly secondary, while their willingness to provide an (expert) opinion becomes the main criterion. Thus, the influence of a few on the content of the JME and the methods seen as acceptable increases. In addition, the burden on the few remaining reviewers increases, potentially further reducing their number and thus exacerbating the problem. 

More than authors, from whom reviewers are usually recruited, every journal has readers. So why don't we ask our readers to review more of the submitted manuscripts? Apart from the obvious advantage that the reviewing process is spread between more people, there are other reasons. For example, we explicitly ask our reviewers at JME to evaluate submitted manuscripts according to the following questions and criteria [2]:

“Topicality/originality/relevance of the article to the reader? For example, is all relevant information contained in the title or summary so that the reader gets a correct picture of the article? Is a clear research question/hypothesis asked or is the aim of the paper explained? Is the study design precisely described? Do graphics/tables correspond to the text? Are the data suitable for answering the research question? Are the data presented in a comprehensible and complete manner? Is the relevance of the results adequately presented? Are the conclusions plausible? Are questions/hypotheses/objectives answered?“ We also ask about readability, expression, grammar, spelling, structuring of the text and comprehensibility.

None of these questions require a thorough knowledge of the relevant literature in the field or of the methods used, as can be expected from other authors, but not necessarily from every reader. Wouldn't it be plausible to have at least one of the expert opinions, of which we typically require two, actually made by a reader, when the majority of our evaluation criteria are already explicitly intended "for the reader"? A very practical problem here: we simply only know from our authors how to contact them.

So my suggestion for a good resolution in 2020: Let the editorial office know how we can reach you and help to further develop the quality and relevance of the JME through constructive and timely reviews. As editors, our decisions can only be improved by a broader perspective - and as reviewers, you are always right [3] anyway: either because you name an actual problem in the article, or because there is no actual problem, but the article is ambiguous or unclear. 

There are numerous sources on what makes good reviews (e.g. this one: https://www.psychologicalscience.org/observer/twelve-tips-for-reviewers) and reviewing yourself will certainly make you a more attentive reader and probably also a more successful author. So it is a little bit like with all good intentions: there is little to say against it. So let’s do it.

## Competing interests

The author declares that he has no competing interests.
